# 8-Methoxypsoralen is a competitive inhibitor of glutathione S-transferase P1-1

**DOI:** 10.3389/fncel.2014.00308

**Published:** 2014-09-30

**Authors:** Diêgo Madureira de Oliveira, Marcel Tavares de Farias, André Lacerda Braga Teles, Manoelito Coelho dos Santos Junior, Martins Dias de Cerqueira, Rute Maria Ferreira Lima, Ramon Santos El-Bachá

**Affiliations:** ^1^Laboratory of Molecular Pathology of Cancer, University of BrasiliaBrasilia, Brazil; ^2^Laboratory of Clinical and Toxicological Analysis, São Rafael HospitalSalvador, Brazil; ^3^Laboratory of Molecular Modeling, State University of Feira de SantanaFeira de Santana, Brazil; ^4^Laboratory of Natural Products, Institute of Chemistry, Federal University of BahiaSalvador, Brazil; ^5^Laboratory of Neurochemistry and Cell Biology, Institute of Health Sciences, Federal University of BahiaSalvador, Brazil

**Keywords:** GST, 8-MOP, glioblastoma

## Abstract

The blood-brain barrier (BBB) is known to protect healthy brain cells from potentially dangerous chemical agents, but there are many evidences supporting the idea that this protective action is extended to tumor cells. Since the process of angiogenesis in brain tumors leads to BBB breakdown, biochemical characteristics of the BBB seem to be more relevant than physical barriers to protect tumor cells from chemotherapy. In fact, a number of resistance related factors were already demonstrated to be component of both BBB and tumor cells. The enzyme glutathione S-transferases (GST) detoxify electrophilic xenobiotics and endogenous secondary metabolites formed during oxidative stress. A role has been attributed to GST in the resistance of cancer cells to chemotherapeutic agents. This study characterized 8-methoxypsoralen (8-MOP) as a human GST P1-1 (hGST P1-1) inhibitor. To identify and characterize the potential inhibitory activity of 8-MOP, we studied the enzyme kinetics of the conjugation of 1-chloro-2,4-dinitrobenzene (CDNB) with GSH catalyzed by hGST P1-1. We report here that 8-MOP competitively inhibited hGST P1-1 relative to CDNB, but there was an uncompetitive inhibition relative to GSH. Chromatographic analyses suggest that 8-MOP is not a substrate. Molecular docking simulations suggest that 8-MOP binds to the active site, but its position prevents the GSH conjugation. Thus, we conclude that 8-MOP is a promising prototype for new GST inhibitors pharmacologically useful in the treatment of neurodegenerative disorders and the resistance of cancer to chemotherapy.

## Introduction

The Brain Blood Barrier (BBB) protects central nervous system (CNS) against chemical and biological insults. It was described as composed mainly of highly specialized endothelial cells with tight junctions, and astrocyte endfeets with anchoring transmembrane proteins (Bentivoglio and Kristensson, 2014). However, nowadays it is clear that a number of enzymes are also part of this barrier (Shawahna et al., 2013). The idea that the BBB has not only a physical constitution, but also a metabolic one, is not new (Minn et al., 1991; El-Bachá and Minn, 1999), drug-metabolizing enzymes continues to be identified (Decleves et al., 2011), and they represent difficulties in drug delivery to the brain (Bentivoglio and Kristensson, 2014). Therefore, BBB is a critical obstacle for the pharmacologic treatment of brain tumors (Jovćevska et al., 2013), leading to research on BBB-disrupting strategies for enhanced drug delivery in these cases (Liu et al., 2014).

Glutathione *S*-transferases (GST; EC 2.5.1.18), multifunctional enzymes which are mainly involved in phase II metabolism and antioxidant cell systems (Di Pietro et al., 2010), are among the drug-metabolizing enzymes present in BBB. It seems that these GSTs, mainly the GSTP1 (GST-π) isoform, which is the most abundant at the BBB, protect cells against oxidative stress and are involved in the phenomenon of drug resistance (Shawahna et al., 2013). In fact, GSTs promote the conjugation between drugs and the tripeptide glutathione (GSH), and special attention has been given to these enzymes since they are strongly associated with resistance, remarkable in cancer (Sau et al., 2010). The most highly expressed GST isoenzyme in various human cancerous and precancerous tissues is also GST-π (Sau et al., 2010). Overexpression of this class of GST was associated with drug resistance or poor prognosis in many kinds of tumors (Wang et al., 2007, 2011; Pasello et al., 2008; Geng et al., 2013), including gliomas (Okcu et al., 2004; Calatozzolo et al., 2005), the most common form of primary brain tumors, and glioblastoma (the highest malignant glioma) (Lo et al., 2004), the most frequent brain tumor in adults (Brennan, 2011). Furthermore, it was documented that GST-π polymorphisms are associated with survival in anaplastic glioma patients; an explanation is that lower activity GST genotypes will allow more prolonged exposure of tumor cells to chemotherapeutic agents (Kilburn et al., 2010).

There are experimental observations suggesting that inhibition of GST increases the response of glioblastoma cells to alkylating agents better than the inhibition of the enzyme O^6^-methylguanine-DNA methyltransferase (MGMT) (Juillerat-Jeanneret et al., 2008), the most frequently associated factor to temozolomide resistance in glioblastomas (Mrugala and Chamberlain, 2008). Therefore, inhibition of Pi-class GST activity or inhibition of its expression has been shown to increase the tumor sensitivity to many drugs (Sau et al., 2010). Indeed, many inhibitors and pro-drugs targeted to GSTs have been developed for a long time, but the clinical effectiveness of these molecules is poor and do not justify therapeutic use (Mahajan and Atkins, 2005). The search for effective GST inhibitors for use in cancer therapy is not recent. Almost two decades ago, clinical studies indicated ethacrynic acid as a candidate for modulation of drug resistance (Lacreta et al., 1994). At the same time, the selective GST-π inhibitor TER-117 was synthesized (Lyttle et al., 1994 include on references), but there are no recent studies with this. Other GST-π specific inhibitors were able to revert multiple drug resistance in cholangiocarcinoma (Nakajima et al., 2003).

In this work we presented the 8-MOP, a well-known drug that has been used for decades in the treatment of skin disease (Tzaneva et al., 2009), as a novel promising molecule to develop new GST-π inhibitors, detailing its mechanism of action, and showing the potential for cancer therapy by using an *in vitro* model of glioblastoma.

## Materials and methods

### Assay of glutathione *S*-transferase activity

GST activity was measured as previously described (van Haaften et al., 2001) with modifications. Briefly, the reaction of 1 mM GSH with 1 mM 1-chloro-2,4-dinitrobenzene (CDNB) catalyzed by GST (GSTP1-1 at 0.1 U/ml or 0.15 mL of human glioma cells lysate in 100 mM potassium phosphate buffer, pH 6.5, at 25°C) was monitored spectrophotometrically by recording the increase in absorbance at 340 nm. Absorbance units were converted to concentration of DNP-SG (the conjugate of GSH and CDNB) as previously described (Mannervik and Guthenberg, 1981). A correction for the spontaneous reaction was made by monitoring formation of DNP-SG in the absence of enzyme. For inhibitory effect analysis, increasing concentrations of 8-MOP (Sigma®) were used. In the test with lysates of glioma cells, GST activity was expressed as percentage of the control group. 8-MOP was dissolved in DMSO, which was present in all groups and did not affect the GST activity. The effect of 8-MOP in spontaneous formation of DNP-SG was also discounted by monitoring the reaction in the presence of the drug and absence of enzyme. To study the inhibitory mechanism, substrate concentrations (CDNB or GSH) were varied (when GSH concentration was varied, the CDNB concentration was kept at 1 mM and vice versa). Five independent experiments were performed with and without 8-MOP at a concentration near the IC_50_. *V*_max_ and *K*_m_ values were calculated by non-linear regression following the Michaelis-Mentem mode and the mechanism was visualized by Lineweaver-Burk plot.

### UV-vis spectrophotometry and high performance liquid chromatography (HPLC) analysis

Electronic absorption spectra of solutions containing CDNB with GSH or 8-MOP with GSH in the presence or absence of GST-π were recorded using a Hewlett-Packard model 8452 A spectrophotometer in 1 cm quartz cuvettes and compared to CDNB alone and 8-MOP alone spectra. Chromatographic analyses of CDNB/CDNB-conjugate and 8-MOP/8-MOP-conjugate were performed according to previous description in literature (Wang and Jiang, 2006; Vaidya and Gerk, 2007) with modifications (Supplementary Table S1). A dual wavelength ultraviolet absorbance detector was used.

### *In silico* approach

A docking study of the 8-MOP in the GST structure (Protein Data Bank code 3IE3) was conducted. In this structure the enzyme is complexed with 6-(7-nitro-2,1,3-benzoxadiazol-4-ylthio)hexanol (NBDHEX) (Federici et al., 2009). The space for molecular docking was defined based on the location of the NBDHEX, as well as the amino acids with important interactions. This space consisted of a 14.0 Å sided box, spaced 1 Å grid. The AutoDock Vina 1.1.2 was used in standard configuration for molecular docking with 8-MOP. The resulting geometry of each enzyme-ligand complex was submitted to a molecular dynamic simulation (MD) using AMBER 10.0 (Case DA, AMBER 10—University of California, San Francisco, 2008). It was performed a two nanoseconds simulation, at 300 K, and with the implicit solvent model described by Hawkins et al. (1996). Separately, protein, 8-MOP, and the NBDHEX structures were submitted to the same MD calculation. The binding energies (E_bind_) were obtained from the average potential energies of the structures resulting from the MD through the equation Ebind = Ecomplex − (EGST + Eligand), in which E_complex_, E_GST_ and E_ligand_ correspond to the average potential energies of the complex ligand-GST, the enzyme alone and the ligand alone respectively. PyMOL (The Molecular Graphics System, 2002—DeLano Scientific, San Carlos, CA, USA) was used for visual interpretation of the results.

### Cell cultures and preparation of cytosolic protein extract

Human glioblastoma GL-15 cells (Bocchini et al., 1991) were cultured at 37°C in Dulbecco modified Eagle's medium (DMEM), supplemented with 1 mM pyruvic acid, 2 mM L-(+)-glutamine, 44 mM NaHCO_3_, 10% fetal bovine serum, 100 IU/mL penicillin and 100 μg/mL streptomycin in a humidified atmosphere of 5% CO_2_ and 95% air. The culture medium was changed every 2 days. Primary cultures of astrocytes from Wistar rats were performed according to previous descriptions (Silva et al., 2008) and maintained under the same conditions described above. The study was conducted according to guidelines of the institutional animal ethics committee (Federal University of Bahia—Brazil). In order to evaluate the activity of GST from GL-15 cells, confluent cultures in 10 mm dishes were lysated with 1 mL of distilled water under vigorous shaking for 30 min. The extract was centrifuged at 5000 g (10 min) and the supernatant stored at −70°C until use in GST activity assays.

### Immunocytochemistry

GST-π expression in GL-15 cells was attested by immunostaining with anti-GSTP1-1 antibody. The cells were permeabilized in methanol at −20°C for 10 min and incubated with the primary antibody (rabbit polyclonal IgG anti GSTP1/2—Santa Cruz®, 1:500) for 1 h. Subsequently, cells were rinsed three times with PBS, incubated with the secondary antibody (Conjugated Alexa Fluor® 546 goat anti-rabbit IgG—Invitrogen®, 1:400) and finally observed by fluorescence microscopy (Olympus AX70 microscope—green filter). Nuclei were stained by the dye Hoechst 33258 (ex/em 340/510 nm) (Oliveira et al., 2010). For negative control, cells were incubated with only secondary antibody under the same conditions described above.

### Evaluation of intracellular reduced glutathione content

Monochlorobimane (MCB) assay (Ublacker et al., 1991) was used to evaluate GSH depletion. After 30 min exposure to 8-MOP (0.05 or 0.4 mM) and CDNB (0.05 mM), GL-15 cells were washed three times with PBS, incubated with 1 mM MCB in medium with 1% ethanol for 40 min, washed again and observed by fluorescence microscopy (Olympus BX 51—URA2, San Jose, USA). The fluorescence mirror unit Olympus U-MWU2 was selected to observe cells (ex/em 330–385/420 nm). The exposure time of 60 ms was used in micrographs for all samples.

### Cell viability measurement and microscopic analysis

In order to evaluate the chemosensitizer potential of 8-MOP, cells were seeded in 96-well plates at a density of 3.1 × 10^4^ cells/cm^2^, and cultured for 24 h prior to treatments with increasing concentrations of chemotherapeutic drugs for 48 h in the presence or absence of 0.05 mM 8-MOP, which was added 2 h before treatments. Both drugs and 8-MOP were dissolved in DMSO (final concentration 0.5% v/v). Cell viability was measured by the 3-(4,5-dimethylthiazol-2-yl)-2,5-diphenyltetrazolium bromide (MTT) method (Mosmann, 1983). In short, after treatment the MTT reagent was added to each well (1 mg/mL). Following additional 2 h incubation, 100 μL of 20% SDS was added. The absorbance was then measured at 595 nm using a microplate reader (THERMO PLATE, model TP-reader—type B). Wells without cells were used as blanks. To access cytotoxicity of 8-MOP, GL-15 cells were treated for 72 h under the same described conditions. Cell growth after long-term (10 days) exposure to a low dose 8-MOP (0.02 mM), added during each medium change, was evaluated by Trypan blue exclusion assay (Louis and Siegel, 2011) in cultures at low cell density (3.86 × 10^3^), and expressed as percentage of cells in the first day. Changes in cell morphology were observed by contrast phase microscopy and nuclear morphology was assessed by Hoechst 33258 staining.

### Statistics analyses

Data were showed as mean with SEM or median with range according to their distribution, analyzed by Shapiro-Wilk normality test and Skewness (normal: < 1 or > −1) and Kurtosis (normal: < 2 or > −2) calculation. Parametric or non-parametric statistic tests were also chosen according to the distribution. The most appropriate test for each experiment was used and this information is in the respective figure legends. At least three independent experiments were done for each assay.

## Results

### 8-MOP inhibits GST-π activity

**Figure 1:**
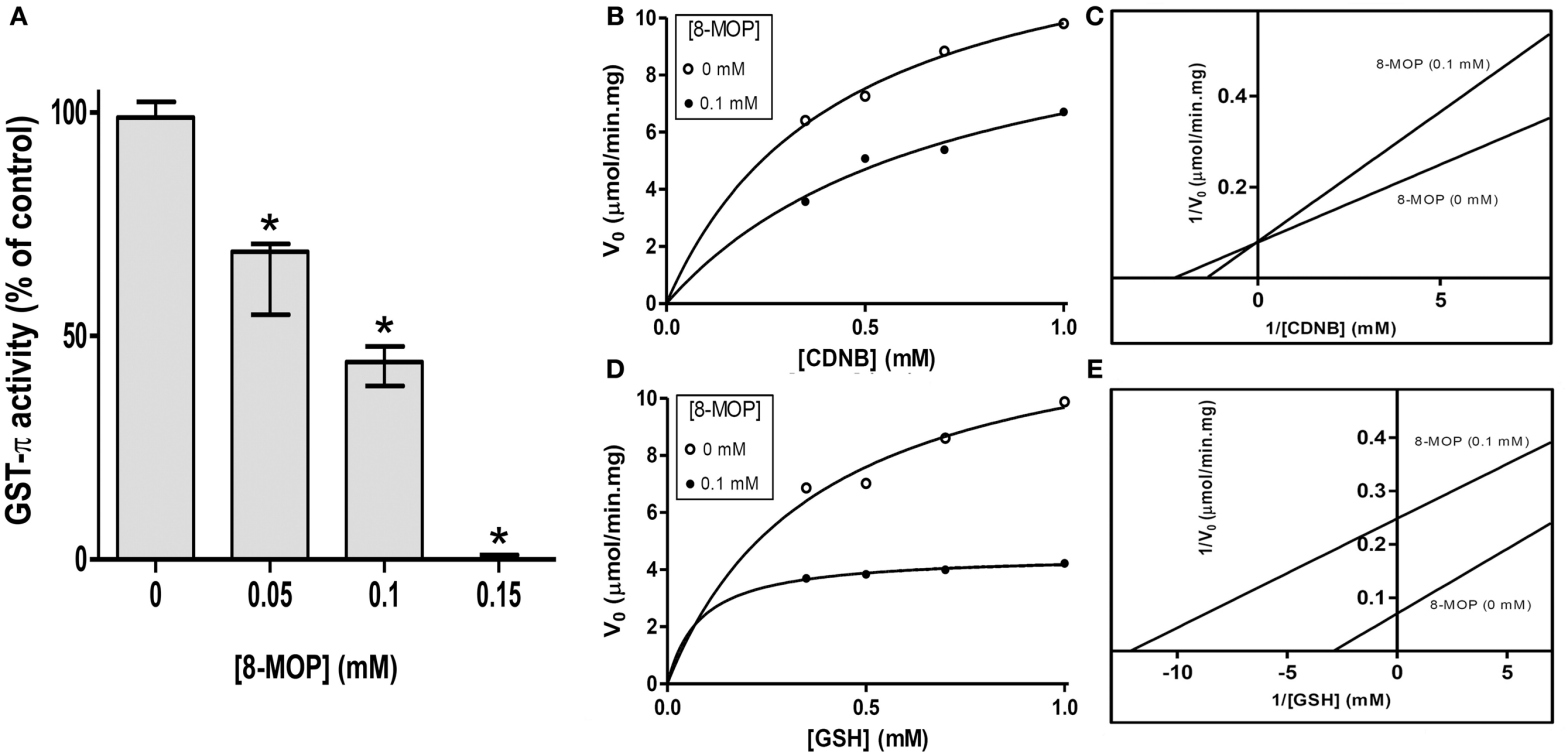
**(A)** Concentration-dependent inhibition of GST-π activity by 8-MOP. Data did not present normal distribution and were analyzed by Kruskal-Wallis non-parametric test followed by Dunn's Multiple Comparison test; ^*^p < 0.05 compared to the control group. **(B)** Michaelis-Mentem (*R*^2^ = 0.9874 without 8-MOP and 0.9545 with 8-MOP) and **(C)** Lineweaver-Burk plots showing competitive inhibition of human GST-π toward CDNB by 8-MOP. The *K*_m_ and *V*_max_ of the enzyme for CDNB were 0.30 mM (*SD* = 0.05) and 11.58 μmol/min.mg (*SD* = 0.77), respectively, but these same values in the presence of 0.1 mM 8-MOP were 0.68 mM (*SD* = 0.06) and 11.71 μmol/min.mg (*SD* = 3.10). **(D)** Michaelis-Mentem (*R*^2^ = 0.9733 without 8-MOP and 0.9736 with 8-MOP) and **(E)** Lineweaver-Burk plots showing an uncompetitive inhibition of human GST-π toward GSH by 8-MOP. The *K*_m_ and *V*_max_ for GSH were 0.25 mM (*SD* = 0.08) and 11.36 μmol/min.mg (*SD* = 1.31), respectively. These values in the presence of 0.1 mM 8-MOP were 0.09 mM (*SD* = 0.02) and 4.70 μmol/min.mg (*SD* = 0.21). The enzyme was used at 0.1 U/mL. The graphs are representative of five independent experiments.

GST activity was concentration dependently inhibited by 8-MOP (Figure 1A), with the IC_50_value of 0.092 mM (Supplementary Figure S1A). The enzyme shows a characteristic Michaelis Menten behavior toward both substrates, but the inhibitor presented a double behavior. A competitive inhibition pattern was observed when 0.1 mM 8-MOP was incubated with GST- π and varying concentrations of CDNB (Figures 1B,C). The presence of 8-MOP increased the calculated *K*_m_ value, but it did not change significantly the calculated *V*_max_ value, indicating that both, inhibitor and substrate (CDNB) bind to the same region of the active site. However, when 0.1 mM 8-MOP was tested varying GSH concentrations both *K*_m_ and *V*_max_ values decreased, which can be interpreted as an uncompetitive inhibition (Figures 1D,E), indicating that inhibitor and substrate (GSH) bind to different sites in the enzyme or different regions of the active site.

### 8-MOP is not a GST-π substrate

**Figure 2:**
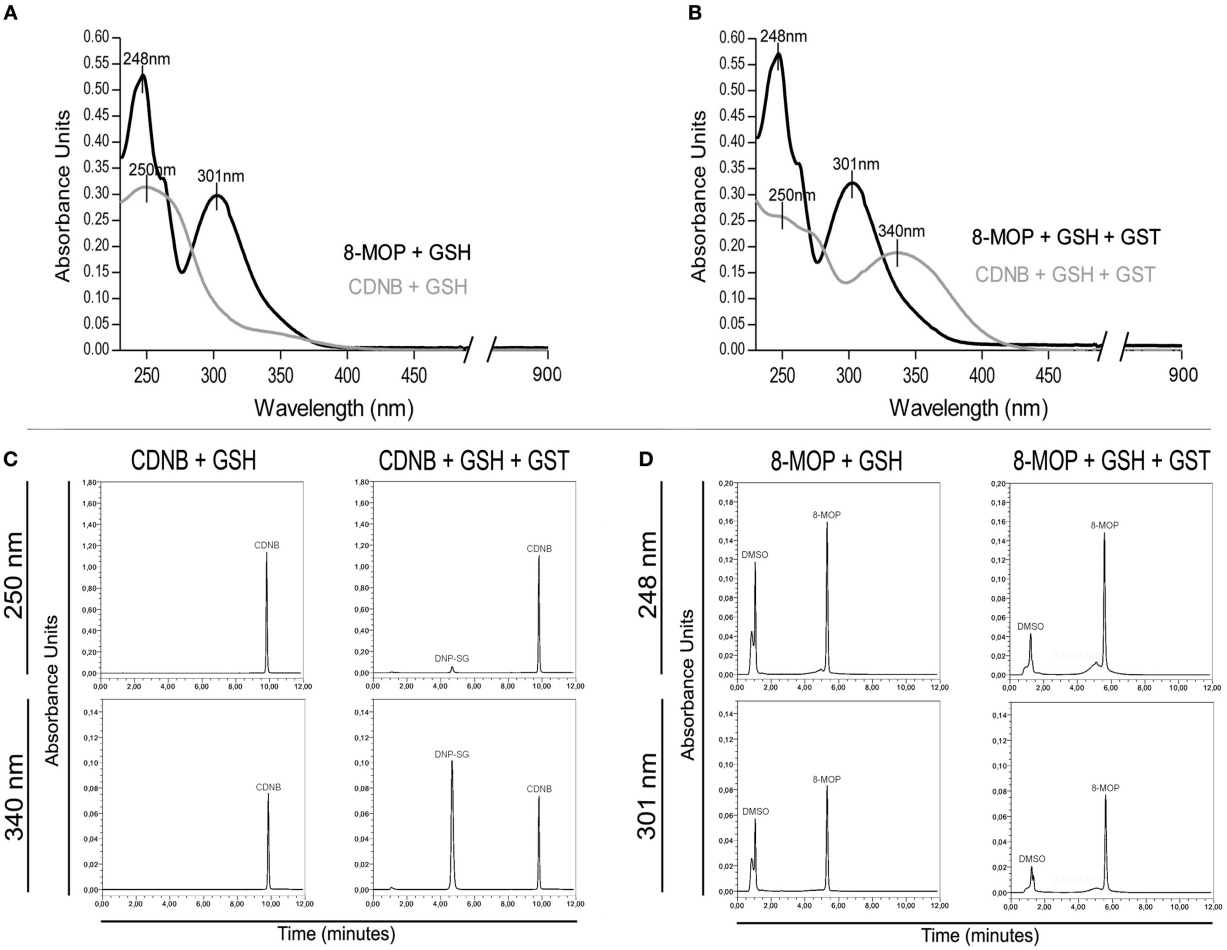
**(A)** Absorption spectra of CDNB and 8-MOP in the presence of GSH. **(B)** Spectrophotometric evidences of DNP-SG, but not “8-MOP-SG,” in spectra after addition of GST-π. The maximum absorbance values for the solutions containing CDNB/GSH/enzyme (250, 340 nm) and 8-MOP/GSH/enzyme (248, 301 nm) were used for HPLC analysis. **(C)** Chromatographic detection of DNP-SG (RT = 4.6 min.), and **(D)** no evidences of “8-MOP-SG.”

Since 8-MOP inhibits competitively GST-π activity, we hypothesized that this drug could be a substrate, just as CDNB. If it was true, a new compound “8-MOP-SG” (Supplementary Figure S1B) would be formed. UV-Vis spectrophotometric analysis clearly showed the generation of DNP-SG that has a different absorption spectrum when compared to CDNB, by addition of GSH in the presence of GST-π. In contrast, the addition of enzyme in the solution containing 8-MOP/GSH did not change its absorption profile, suggesting no alteration in the 8-MOP structure (Figures 2A,B). To certify that 8-MOP-SG was not present in the solution, HPLC was carried out using maximal absorbance values for each solution for detection. Again, DNP-SG was identified with a retention time (RT) lower than CDNB (Figure 2C), but a single peak was present in the chromatogram for 8-MOP/GSH plus GST-π (Figure 2D). The theoretical log *P*-value for CDNB and log D value for DNP-SG are 2.46 and −3.14, respectively (Supplementary Figure S2), which justifies the lower RT of DNP-SG. On the other hand, the log *P*-value for 8-MOP is 1.78 and the theoretical log D value for the proposed 8-MOP-SG is −2.58, but no peak in a very low RT was visualized in the chromatogram. The absorption spectrum and chromatographic profiles were the same even after 30 days incubation (data not shown). Therefore, these data support the idea that 8-MOP-SG is not formed or it is formed in a very low extent.

### 8-MOP efficiently interact with the active site of the enzyme

**Figure 3:**
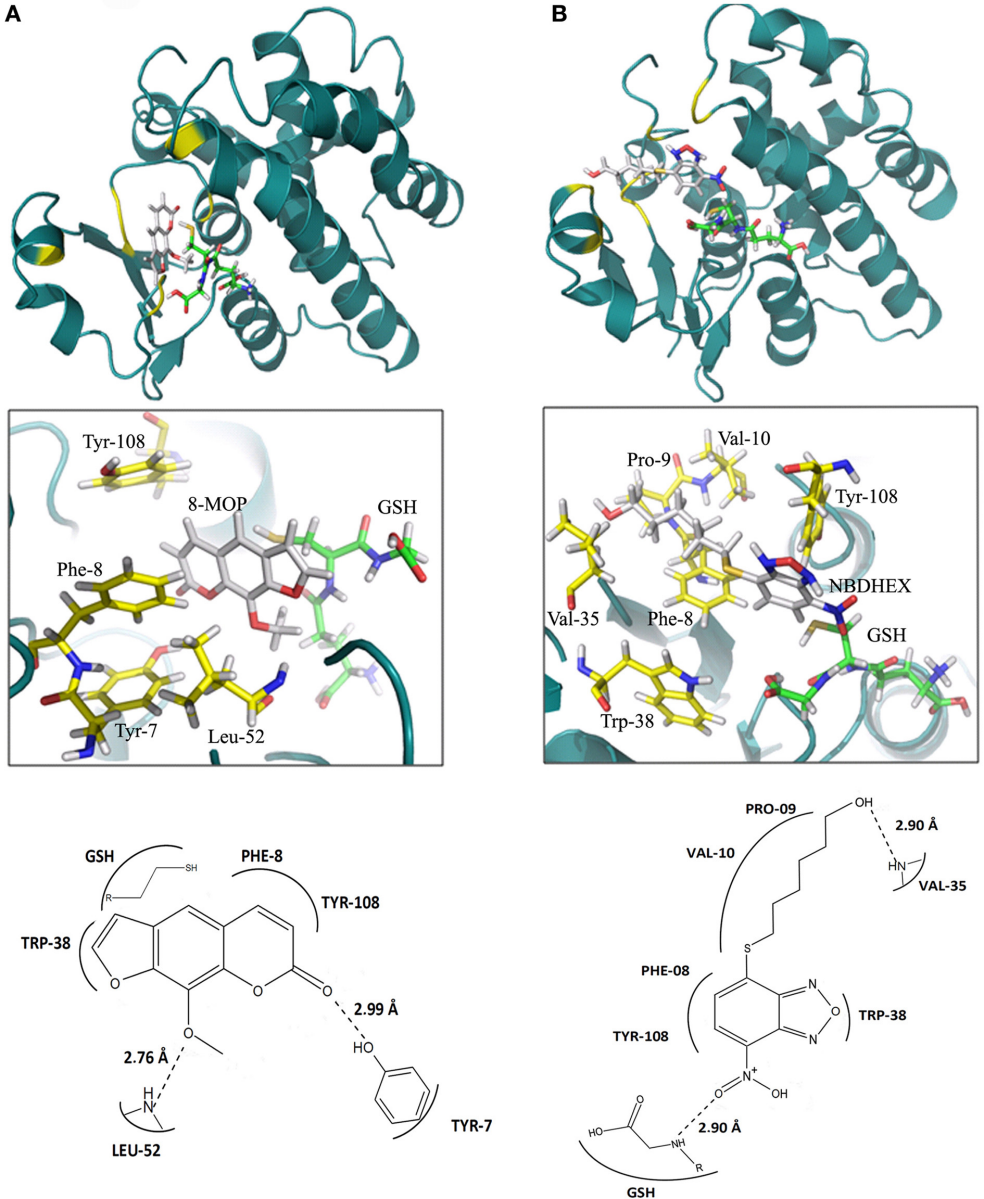
**(A)** Interactions between 8-MOP (white) and GST after 2 ns simulation. The regions of interactions are in yellow. It is also showed details (interacting residues in yellow) and scheme of interactions between residues in the active site of GST and the 8-MOP. GSH is in Green. **(B)** The same for the inhibitor NBDHEX.

*In silico* data strongly suggest an efficient GST-π activity inhibition by 8-MOP that binds to the active site of the enzyme. The score obtained with Auto Dock Vina as well as the calculations of binding energy from the MD showed good stability of 8-MOP when compared with NBDHEX (Table 1). Hydrophobic interactions are made by 8-MOP coumarin core with residues Phe-8 and Tyr-108. Moreover, it is clear that the geometric position of 8-MOP inside the active site prevents the 8-MOP-SG formation (Figure 3A). In addition, 8-MOP makes another important interaction with Trp-38 and forms hydrogen bonds with Tyr-7 and Leu-52. These two last residues apparently do not directly interact with the NBDHEX. However, the benzoxadiazole ring of this inhibitor makes the same hydrophobic interactions with residues Phe-8 and Tyr-108 observed in 8-MOP/GST interactions (Figure 3B). The redocking of the inhibitor NBDHEX in the GSTP1-1 by AutoDock Vina presented a Root Mean Square Deviation (RMSD) of 1.99 Å from the respective crystal structure (Supplementary Figure S3A), which is an acceptable deviation docking value. Furthermore, RMSD vs. time graphics (Supplementary Figures S3B,C) showed less pronounced variation for the 8-MOP complex, which could indicate an effective stabilization of the system by 8-MOP.

**Table 1:** **Energies from the docking of 8-MOP and NBDHEX**.

**Complexes**	**AMBER**				**Autodock Vina (Kcal/mol)**
	**Average potential energies (Kcal/mol)**			**Binding energy (Kcal/mol)**	
	**Complex**	**Ligand**	**GST**		
**NBDHEX/GST**	−6131.88	41.73	−6136.48	**−37.13**	**−5.4**
**8-MOP/GST**	−6697.87	15.87	−6136.48	**−577.26**	**−6.4**

### 8-MOP inhibits GST from tumor cells and is not substrate for other isoforms of GST

**Figure 4:**
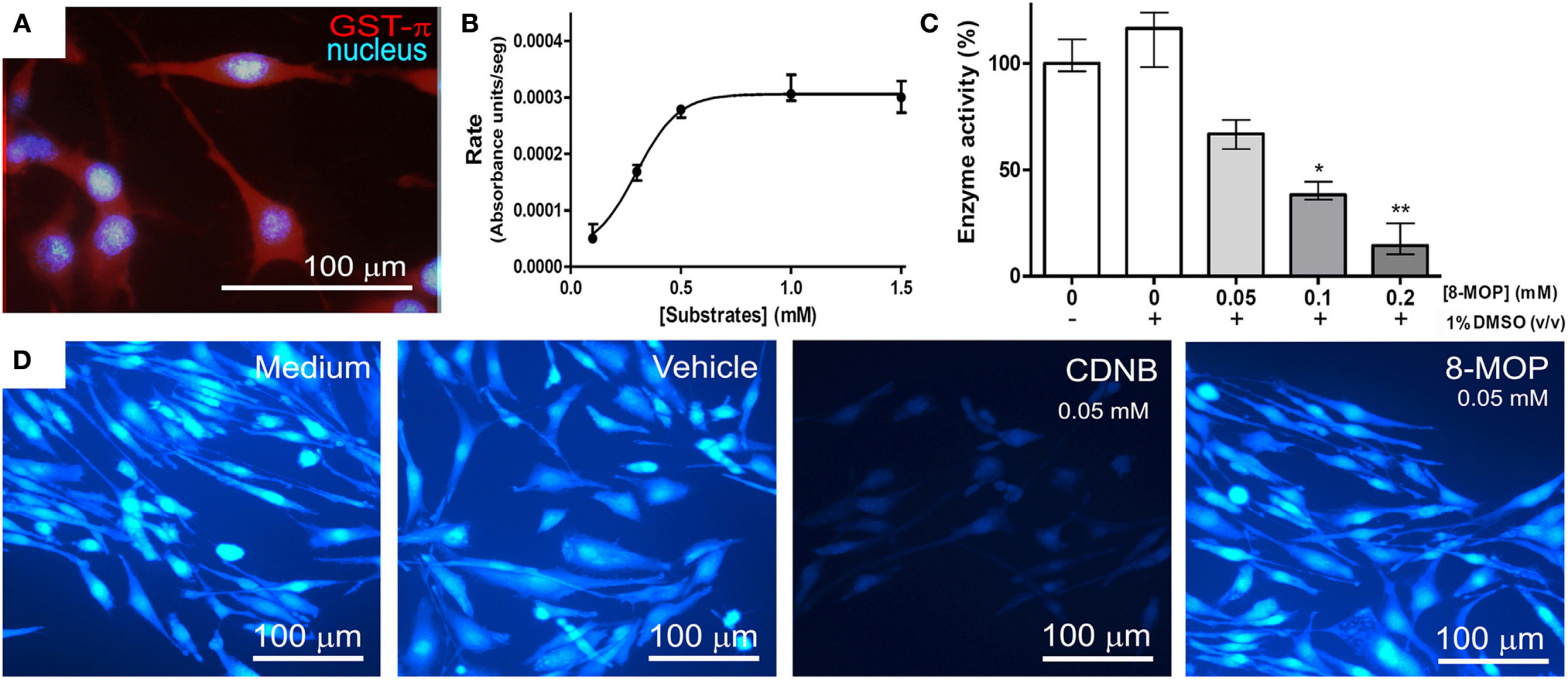
**(A)** GL-15 cells labeled positively to GST-π. **(B)** Conjugation reaction between CDNB and GSH under increasing concentrations catalyzed by GST in the cytosolic protein extract of GL-15 cells, followed until the maximum rate was attained. Line represents non-linear regression (*R*^2^ = 0.9770) of the data. **(C)** Concentration-dependent inhibition of GST by 8-MOP under maximum rate conditions (1 mM CDNB). Data did not present normal distribution and were analyzed by Kruskal-Wallis non-parametric test (excluding the first group without DMSO) followed by Dunn's Multiple Comparison test ^*^p < 0.05 and ^**^p < 0.01 compared to the control group (1% DMSO, without 8-MOP). **(D)** Intracellular GSH assessed by MCB assay. GSH was present in control conditions and was depleted by CDNB after 30 min exposure, but not by 8-MOP.

GST activity in GST-π positive tumor cells (Figure 4A) was investigated. *K*_m_ and *V*_max_ calculation could not be performed since there was not only one isoform of GST in the lysate. Then, data were analyzed by non-linear regression (*R*^2^ = 0.9770) (Figure 4B). Substrate concentrations greater than 0.5 mM saturated the amount of enzyme present in the volume of lysate used (0.15 mL), and saturating conditions (substrate at 1 mM) were used to investigate GST activity inhibition by 8-MOP, which showed a concentration-dependent pattern (Figure 4C). Additionally, treatment with 0.05 mM CDNB for 15 min depleted intracellular reduced GSH, as expected, but 8-MOP did not promote GSH depletion (Figure 4D), even at 0.4 mM (data not shown), giving support to our hypothesis that 8-MOP does not conjugate with GSH. The addition of protein extract from tumor cells did not also change the spectrum of 8-MOP/GSH solution (data not shown).

### 8-MOP sensitizes glioblastoma cells to drugs and presents intrinsic antitumor effect

**Figure 5:**
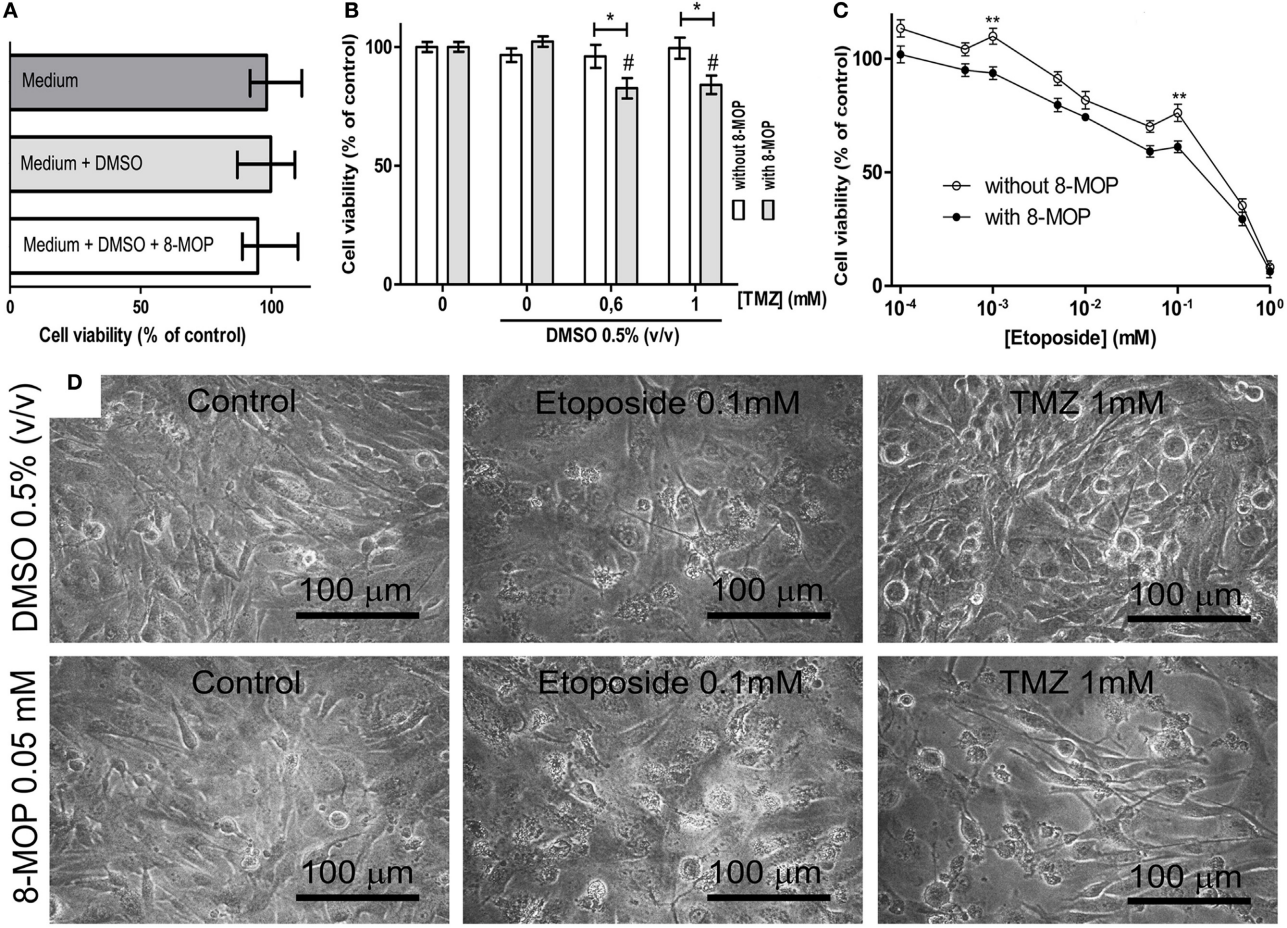
**(A)** MTT assay data showing that treatment with vehicle and 8-MOP did not change cell viability. **(B)** GL-15 cells become more sensible to high concentrations of temozolomide (TMZ) after treatment with 8-MOP, that significantly reduced viability (MTT assay) of 0.6 and 1 mM TZM treated cells in 17.4% and 16%, respectively. **(C)** 8-MOP also acted as chemosensitizer for etoposide, what was statistically significant at 0.001 and 0.1 mM. DMSO was kept at 0.05% and 8-MOP at 0.05 mM in these experiments. ^*^p < 0.05 and ^**^p < 0.01. ^#^*p* < 0.01 compared to group treated with 8-MOP alone in the experiment with TMZ (Two-way ANOVA and Bonferroni post-test). Values are means (±s.e.m.). **(D)** The pictures show reduction of cellularity and morphological changes promoted by the associations.

**Figure 6:**
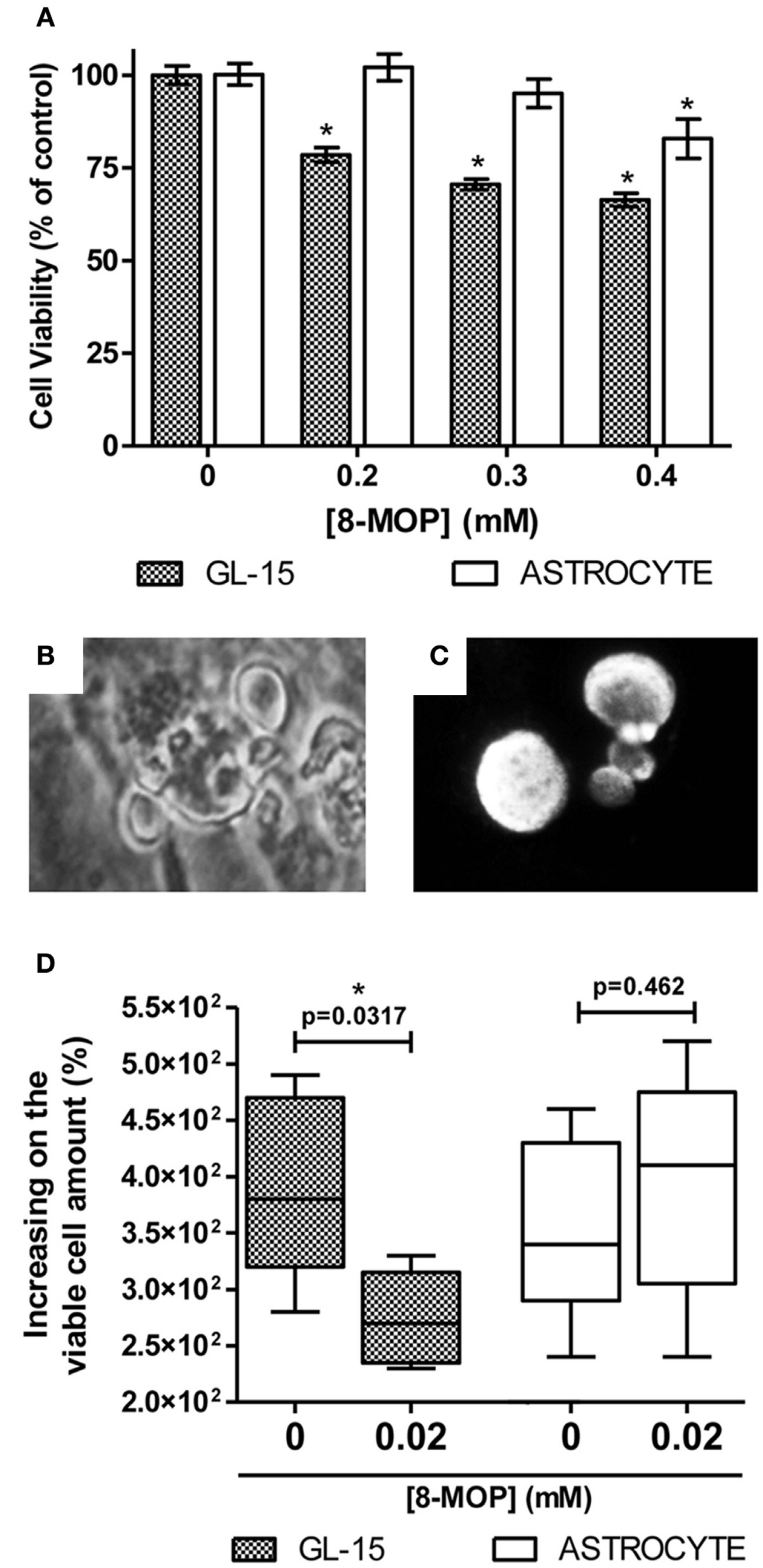
**(A)** Decrease of 21.5% (± 1.9), 29.4% (± 1.4) and 33.4% (± 1.8) on GL-15 viability after 48 h treatment with 8-MOP at increasing concentrations (gray bars). Normal cells (white bars) were less affected (decrease of 17.2% (± 5.3) only at maximal concentration). Data are from MTT assay. Values are means (±s.e.m.). ^*^p < 0.01 (One-way ANOVA and Dunnett's Multiple Comparison post-test, data from each cell type were statistically analyzed separately). 0.05% DMSO was present in all groups. **(B)** Cell blebbing visualized by phase contrast microscopy and **(C)** condensed/fragmented Hoechst 33258 stained nuclei of GL-15. These findings are suggestive of apoptosis. No morphological evidences of apoptosis were found among treated astrocytes. **(D)** Reduced cell proliferation (evaluated by trypan blue exclusion assay) in GL-15 cells after long-term exposure to low dose of 8-MOP (10 days, 0.02 mM). The number of cells increased 380% (320,470) in control conditions against 270% (235,315) in 8-MOP treated group. Values are median (25% percentile, 75% percentile). Astrocytes were not affected at all. ^*^p < 0.05 (Mann Whitney non-parametric test).

To investigate evidences of the chemosensitizer action of 8-MOP, cells from human glioblastoma (GL-15) pre-treated with a sub-toxic concentration of 8-MOP or vehicle (Figure 5A) were exposed to chemotherapeutic agents. High concentrations of temozolomide lead to decreases of cell viability greater than 15% (Figure 5B) in these cells those are totally resistant to this agent (Supplementary Figure S4). 8-MOP also increased susceptibility to etoposide (Figure 5C), which is a recognized substrate for GST-π. The effects, accessed by MTT assay, were clearly visualized by phase contrast microscopy that shows reduction of cellularity and morphological changes promoted by the associations (Figure 5D). Besides chemosensitizing activity, 8-MOP presented direct and selective *in vitro* antitumor effect. It promoted significant reduction of cell viability in tumor cells, but rat astrocytes used as normal cells control were not affected to the same extent (Figure 6A). Microscopic findings suggest that 8-MOP promotes apoptosis in GL-15 cells (Figures 6B,C). A low concentration of this drug was also effective for reducing cell proliferation accessed by trypan blue assay after 10 days exposition (Figure 6D). No apoptotic signals were found in this experimental design.

## Discussion

The overexpression of GSTs in many kinds of tumors encourages studies with agents able to promote down regulation of these enzimes (Zhao et al., 2011), GST-actived pro-drugs aiming selective action (Pezzola et al., 2010; Johansson et al., 2011; Kogias et al., 2011) and GSTs inhibitors aiming overpass drug resistance (Cui et al., 2008; Tentori et al., 2011). However, nowadays, there is no GST-based approach consistent enough to be adopted as clinical adjuvant therapy. Ethacrynic acid was the first GST inhibitor, acting as substrate of some isoenzymes of GST, it was utilized to sensitize cancer cells to the cytotoxic effect of alkylating agents. However, a number of substantial side effects such as a marked diuresis have discouraged the use of this molecule in clinical practice (Quesada-Soriano et al., 2011). In this work, we presented 8-methoxypsoralen (8-MOP) as a promising GST-π inhibitor; it is a known drug that has been orally and topically used for decades in the treatment of skin disease like psoriasis and eczema (Tzaneva et al., 2009).

8-MOP concentration-dependently reduced human GST-π activity *in vitro* and also decreased total GST activity in cytosolic extract of human glioblastoma cells. To access the mechanism of action, enzyme kinetic assays were performed and showed a competitive pattern of inhibition with 8-MOP occupying the hydrophobic binding site (H-site) of the enzyme, keeping the glutathione binding site (G-site) free. Since inhibition is competitive, 8-MOP could simply act as a substrate. However, spectrophotometric and chromatographic analyses showed no formation of the 8-MOP/GSH conjugate. Additionally, *in silico* tests showed that 8-MOP alone interacts with the active site more favorably than the inhibitor NBDHEX.

Finally, the results from experiment with monochlorobimane (MCB) suggest that 8-MOP does not act as substrate of GST from any class, since no GSH depletion occurred at all. Whereas conjugation of MCB with GSH is catalyzed by many cytosolic GSTs (Eklund et al., 2002), the MCB assay also reflects the GST activity (Ublacker et al., 1991), and then the results also confirm the reversible nature of the inhibition (since after washing it is possible to visualize fluorescence) what suggests that 8-MOP does not inhibit all cytosolic GSTs, presenting any selectivity.

The NBDHEX is a promising non-GSH peptidomimetic GST-π inhibitor (Ricci et al., 2005) that increased temozolomide efficacy in an *in vivo* model of malignant melanoma (Tentori et al., 2011) and were also effective in overcoming drug resistance in osteosarcoma cell lines (Wang et al., 2007). 8-MOP is a low molecular weight hydrophobic compound that easily crosses the cell membrane; it is also a non-peptidomimetic GST inhibitor, characteristics that made NBDHEX a promising new therapeutic possibility. Docking simulations suggested that 8-MOP interact with active site of GST-π better than NBDHEX. In fact, both, the free form of 8-MOP and the GSH conjugated form had better values of binding energy than NBDHEX. Important hydrophobic interactions made by the benzoxadiazole ring of the NBDHEX with residues Phe-08 and Tyr-108 can also be observed with the 8-MOP coumarin core. These residues are essential for the activity of two other inhibitors: clorambutil (Parker et al., 2008) and ethacrynic acid (Oakley et al., 1997). The 8-MOP also realizes important interactions with Trp-38, Tyr-7, and Leu-52. These residues apparently don't interact with the NBDHEX, as well as the two others inhibitors cited, clorambutil and ethacrynic acid. Furthermore, 8-MOP is structurally simple, what facilitates its synthesis.

Another advantage of 8-MOP is the possibility of short-term clinical using. There are a number of clinical trials with 8-MOP, that is already clinically used (Berroeta et al., 2010), and its toxicity in humans is well known (Sehgal, 1975). Also, many neurological effects of 8-MOP, as insomnia, headache and dysosmia (Vernassière et al., 2006) suggest that this substance is able to cross the blood-brain barrier. However, it is important to consider that some GST genes present polymorphism, including GSTP1 gene for which three variants (hGSTP1^*^A, hGSTP1^*^B, and hGSTP1^*^C) were described. The hGSTP1^*^C variant being expressed at a higher frequency in gliomas than in normal cells (Kilburn et al., 2010; Backos et al., 2012); this variant contains an A-to-G transition, causing an isoleucine-to-valine change and a C-to-T transition, resulting in an Ala114-Val114 (A114V) substitution. These transitions have impact in the catalytic activity of the enzyme (Lang et al., 2012), and then, 8-MOP could act differently in it. Another indication of the influence of GST variant on 8-MOP activity is that GST polymorphism influences the outcome of therapy with 8-MOP, although plasma concentrations of the drug have not been associated with different genotypes of the enzyme (Ibbotson et al., 2012). A future clinical use of 8-MOP as GST inhibitor must take these information into account. Associations between GSTP1 polymorphism (and activity) and other pathological conditions, mainly neurodegenerative diseases, like multiple sclerosis (Alexoudi et al., 2014), Parkinson's disease (Longo et al., 2013) and essential tremor (Martínez et al., 2008), could suggest side effects of inhibition of GST activity, but, in the other hand, they also increase the potential therapeutic scope of this prototype.

Co-expression of GST-π and the efflux pump MRP1 (multi-drug resistance protein 1) was associated with resistance to etoposide (Depeille et al., 2005). 8-MOP was tested as chemosensitizer in cultures of human glioblastoma cells when co-administered with this drug and the standard agent TMZ, and it was effective for both, what is probable due to its GST-π inhibitory action. 8-MOP also showed intrinsic antitumor effect against these cells. Indeed, GST-π is also involved in the regulation of apoptosis through the inhibition of the c-Jun-N-terminal kinase (JNK) signaling pathway. The enzyme binds to JNK preventing its phosphorylation and, hence, blocking its kinase activity (Tew and Townsend, 2011); the oligomerization of GST-π promotes the uncoupling and releases JNK, that can act on apoptotic pathway and amplify cell death stimuli (Laborde, 2010). That is the probable explanation for resistance toward drugs which are not substrates for GSTs in tumor cells those overexpress this enzyme (Tew and Townsend, 2011). In fact, intrinsic proapoptotic activity was also described to other GST inhibitors (Turella et al., 2005; Tregno et al., 2009). However, it is important take into account that the oligomerization of GST-π also influences (negatively) the catalytic activity of the enzyme (Bernardini et al., 2000) and several GST inhibitors, including ethacrynic acid, can promote this oligomerization (Sánchez-Gómez et al., 2013). Thus, the antitumor effect and probable apoptosis promoting activity of 8-MOP is now under investigation.

In conclusion, we have shown new application for a well-established drug. 8-MOP can represent a molecule of a novel class of GST inhibitors. The therapeutic potential of these inhibitors is not restricted to cancer treatment, they could also have application in treatment of infectious diseases since GST activity has been reported in many pathogenic parasites (Mahajan and Atkins, 2005). Ours results also have implications for current treatment using 8-MOP, like PUVA (psoralen plus ultraviolet A) therapy, and neurodegenerative diseases involving GSH depletion or any pathologic condition in which GST has an important role.

### Conflict of interest statement

The authors declare that the research was conducted in the absence of any commercial or financial relationships that could be construed as a potential conflict of interest.
