# Supplementary material for: 8-Methoxypsoralen is a competitive inhibitor of glutathione S-transferase P1-1
Source: Front Cell Neurosci. 2014 Sep 30;8:308. doi: 10.3389/fncel.2014.00308 (PMC4179718; doi:10.3389/fncel.2014.00308)
Supplement: Supplementary file 1 [file DataSheet1.PDF]

# *Supplementary Material*

# 8-Methoxypsoralen is a competitive inhibitor of glutathione S-transferase P1-1

**Diêgo Madureira de Oliveira[1]*, Marcel Tavares de Farias[2], André Lacerda Braga Teles[3], Manoelito Coelho dos Santos Junior[3], Martins Dias de Cerqueira[4], Rute Maria Ferreira Lima[5], Ramon Santos El-Bachá[5]**

[1]Laboratory of Molecular Pathology of Cancer, University of Brasilia, Brasilia, Federal District, Brazil
[2]Laboratory of Clinical and Toxicological Analysis, São Rafael Hospital, Salvador, Bahia, Brazil
[3]Laboratory of Molecular Modeling, State University of Feira de Santana, Feira de Santana, Bahia, Brazil
[4]Laboratory of Natural Products, Institute of Chemistry, Federal University of Bahia, Brazil
[5]Laboratory of Neurochemistry and Cell Biology, Institute of Health Sciences, Federal University of Bahia, Brazil

*** Correspondence:** Diêgo Madureira de Oliveira, PhD. Faculty of Ceilândia, University of Brasilia (UnB); 72220-140, Brasilia, Federal District, Brazil Salvador, Bahia, Brazil. Tel.: +55 61 85756904. dmadureira@unb.br

## Supplementary Figures and Tables

**Supplementary Table S1 -** Gradient elution for chromatographic determination of 8-MOP in solution

| Time (min) | ACN (%) | MeOH (%) | $H_2O$ (%) |
|---|---|---|---|
| 0 | 5 | 15 | 80 |
| 2 | 5 | 30 | 65 |
| 3 | 5 | 70 | 25 |
| 6 | 5 | 15 | 80 |

Detection was performed simultaneously at 248 and 301 nm. For CDNB/DNP-SG, 1% acetic acid and acetonitrile (80:20, v/v) was used as the mobile phase and detection was performed simultaneously at 280 and 340 nm. The flow rates were kept at 1 mL/min. The Alliance® HPLC system 2695 separations module with the 2487 dual wavelength ultraviolet absorbance detector was used. Data were collected and analyzed by the Empower 2 chromatography data software (Waters). A 3.9 x 150 mm x 5 µm C18 column (XTerra MS - Waters) and a 3.9 x 20 mm pre-column (C18, XTerra MS - Waters) were used. The samples were pre-filtered through cellulose membrane (0.45 µm) and the column temperature was maintained at 25°C.

**Supplementary Figure S1**

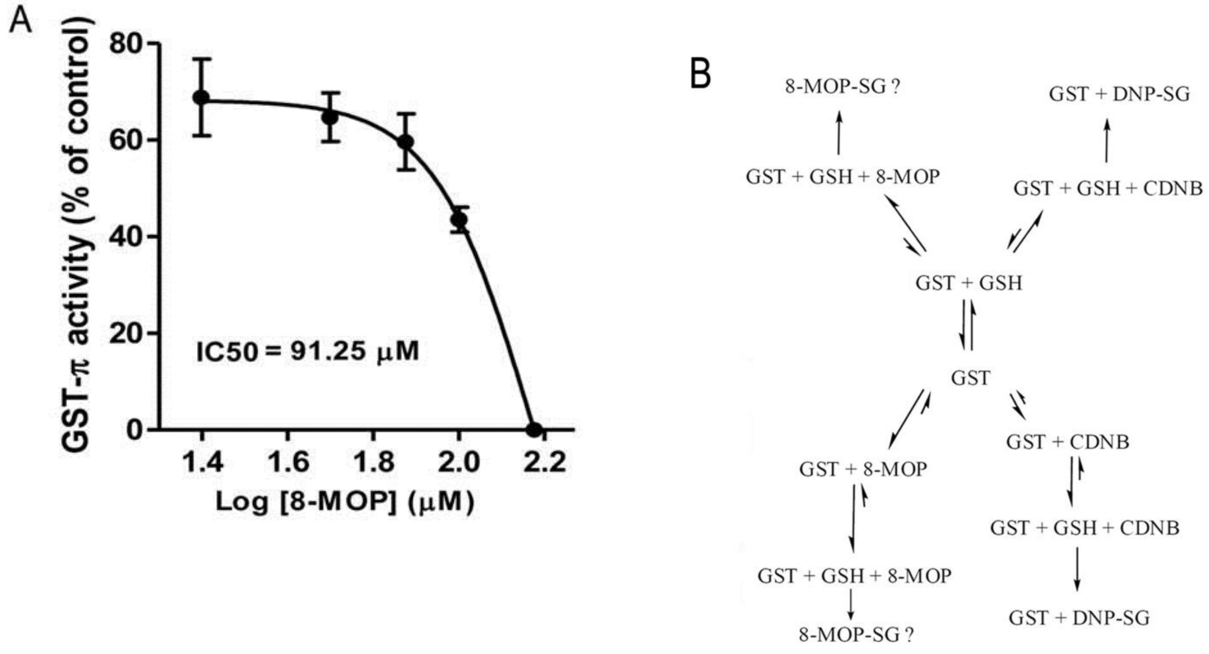


**(A)** Non-linear regression for dose-dependent inhibition of GST-π activity by 8-MOP. The inhibitory effect was fitted to Eq. $A = \{137.97/[1+10^{(3.73LogC-8.14)}]\}-69.72$ in which A corresponds to GST-π activity (in percentage of control) and C is the 8-MOP concentration. The calculated $IC_{50}$ (concentration of 8-MOP able to inhibit 50% of GST-π activity) was 0.092 mM. **(B)** Reaction dynamics scheme showing both competitive and uncompetitive inhibition of human GST-π towards CDNB and GSH, respectively, by 8-MOP. GST activity was concentration dependently inhibited by 8-MOP (Fig. 2a).

**Supplementary Figure S2**

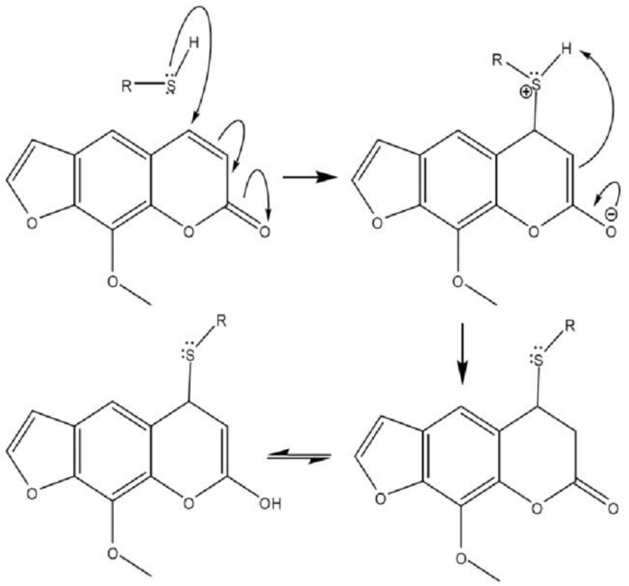

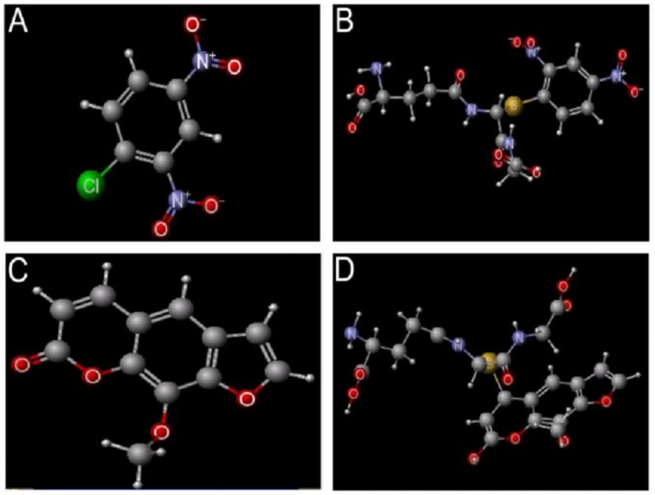


| | CDNB (A) | DNP-SG (B) | 8-MOP (C) | “8-MOP-SG” (D) |
|---|---|---|---|---|
| log P of nonionic specie | 2.46 | -0.74 | 1.78 | -0.17 |
| log D at i.p | | -3.14 | | -2.58 |

Mechanism of reaction[a] proposed to formation of the compound 8-MOP-SG and molecular modeling for theoretical log P calculation[b] for CDNB, 8-MOP, DNP-SG and the supposed 8-MOP-SG. The conjugation with GSH lowered the log P values, as expected, resulting in increased water solubility (i.p. = isoeletric point).

[a] The business end of glutathione is the thiol (SH) group, which is added to the β-carbons in carbonyl compounds by Michael addition. Molecules present absorption peak on ultraviolet radiation only if the energies of possible electronic transitions are in the same order of magnitude as the UV region. As the extent of conjugation increases, decreases the energy difference between HOMO (highest occupied molecular orbital) and LUMO (lowest unoccupied molecular orbital), thus increasing the range of wavelengths in the spectrum in the UV region. The psoralen is conjugated system in a ring that extends to the aromatic furan ring, including the carbonyl group. With the addition of glutathione, via the sulfur atom, the conjugated system involving the furan ring is lost; affecting the energy of the orbitals involved in the length of links, hence there is a change in the range of absorption in the UV, which was not observed in our experiments.

[b] Structures were designed in 2D and converted to 3D by using the softwares ChemDraw 10.0 and Chem3d 10.0, respectivaly. Theoretical log P and log D values were calculated using on-line tool LogP Calculator available in the University of Massachusetts Boston General Biology web site (http://intro.bio.umb.edu/111-112/OLLM/111F98/newclogp.html). For calculation, the method weighted was chosen and the following condition was admitted: $[Cl^-] = [Na^-] = [K^-] = 0.1$ mol/dm$^3$.

**Supplementary Figure S3**

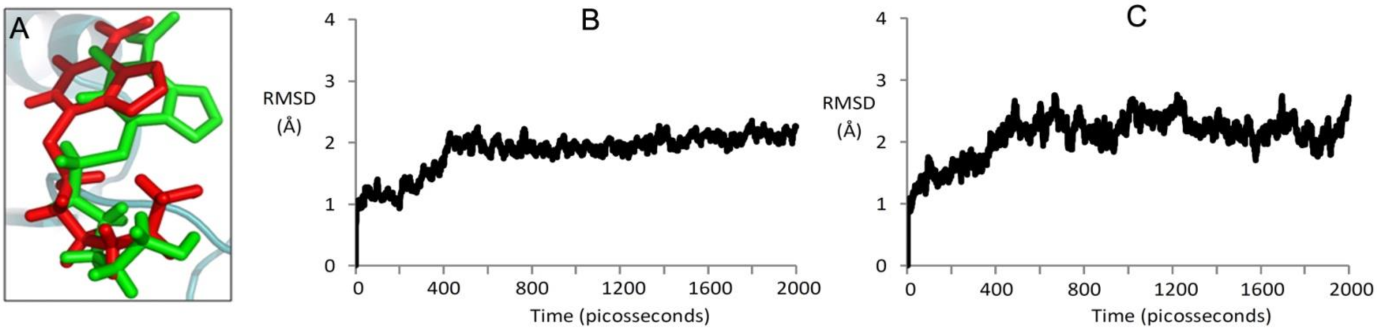


**(A)** Redocking: The overlap of the geometries for NBDHEX in the active site of GST obtained by AutoDock Vina on the crystal structure. In red: experimental geometry. In green: AutoDock Vina docking geometry. RMSD: 1.99 Å. **(B)** Graphics of Root Mean Square Deviation (RMSD) versus time of simulation for the complexes 8-MOP/GST and **(C)** NBDHEX/GST.

**Supplementary Figure S4**

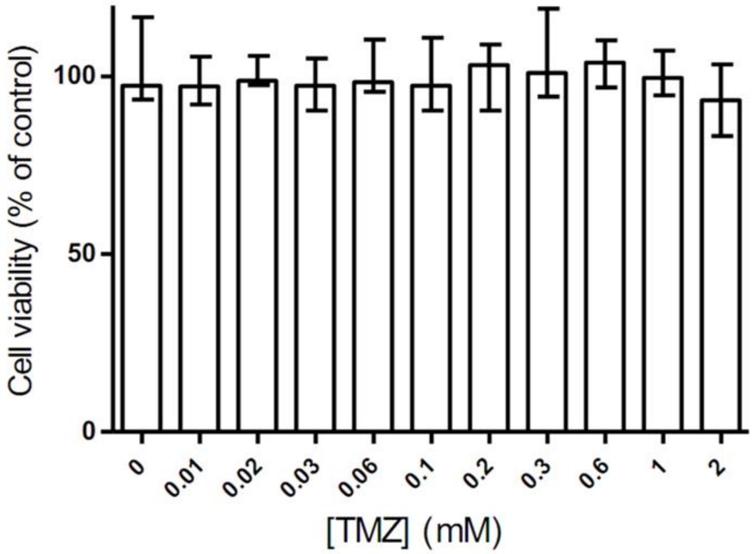


Cell viability (assessed by MTT assay) of GL-15 cells after 72 hours of treatment with TMZ at increasing concentrations. The cells presented extreme resistance toward the drug even at the high concentration of 2 mM. The vehicle (DMSO 0.5% v/v) is present in all groups, including control, but did not affected cell viability (data not shown).
